# Case Report and literature review: complications of the association between rheumatoid arthritis and tuberculosis

**DOI:** 10.3389/fmed.2026.1971306

**Published:** 2026-10-07

**Authors:** Alexandra-Maria Cristea, Dragos-Cosmin Zaharia, Oana Andreea Parliteanu, Ioan Berila, Alexandra Minca, Dragos Minca, Stefan Dumitrache-Rujinski, Ionela Alina Croitoru, Ana Maria Zaharie, Adelina Afetelor, Irina Ruxandra Strambu

**Affiliations:** 1 Carol Davila University of Medicine and Pharmacy, Bucharest, Romania; 2 Marius Nasta Institute of Pneumophtisiology, Bucharest, Romania; 3 Titu Maiorescu University of Medicine and Pharmacy, Bucharest, Romania; 4 Prof. Dr. Agrippa Ionescu Emergency Clinical Hospital, Bucharest, Romania; 5 Dr. Ion Stoia Clinical Center of Rheumatic Diseases, Bucharest, Romania

**Keywords:** arthritis (including rheumatoid arthritis), pneumomediastinum, pneumothorax, subcutaneous emphysema, tuberculosis

## Abstract

**Background:**

Pulmonary tuberculosis is one of the major public health issues globally. According to the World Health Organization, it is among the top 10 causes of death caused by a single pathogen. In Romania, its reported incidence rate in 2024 was approximately 60 per 100,000 people, making it the country with the highest incidence in Europe. Rheumatoid arthritis (RA) is a systemic chronic autoimmune inflammatory disease characterized by the destruction of the small joints along with symmetrical distribution and multiorgan involvement. Pulmonary manifestations are more common in males with positive serology and can involve the lungs, airways, or pleura. Short-term corticotherapy may be initiated in patients with severe symptoms, and biological therapy (anti-TNF alpha drugs) may be used in those with inefficiency of disease-modifying antirheumatic drugs, with an increased risk for disseminated forms of tuberculosis in those with latent infection.

**Case description:**

We present the case of a 38-year-old male patient with a 5-year history of seropositive RA who was treated with methotrexate and methylprednisolone, without any prior investigations for latent tuberculosis infection or any chest radiography performed, and was admitted with severe respiratory failure. The results of thoracic computed tomography showed bilateral pneumothorax and pneumomediastinum, with pulmonary lesions suggestive of a diagnosis of tuberculosis, which was confirmed by bacteriological testing of samples.

**Conclusion:**

The coexistence of two severe diseases—one requiring immunosuppressive treatment and the other one favored by immunosuppression—in a country with a high prevalence of tuberculosis infection and in the absence of latent tuberculosis infection screening or periodic pulmonological assessment can lead to severe, life-threatening complications that require complex, multidisciplinary, long-term, and expensive management.

## 1 Introduction

Rheumatoid arthritis (RA) is a chronic inflammatory disease that affects the joints and is characterized by pain, swelling, redness, stiffness, and tenderness of the joints, which are typically symmetrical. Pathophysiologically, it is an autoimmune disease with increased levels of autoimmune complexes produced by synovial and plasmatic cells (1). The purpose of treatment is to reduce symptoms, prevent the functional and structural damage of the joints, and provide a good quality of life. The drugs used for treatment are disease-modifying antirheumatic drugs (DMARDs), such as methotrexate, biological therapy (bDMARDs: TNF alpha inhibitors, T-cell costimulation modulators, interleukin 6 receptor antagonist, and anti–B cell agents), and targeted synthetic DMARDs (tsDMARDs; Janus kinase inhibitors). European League Against Rheumatism guidelines recommend methotrexate or other csDMARDs as a first-line therapy and biological and tsDMARDs as a second-line therapy in case of a lack of response to the first-line treatment, because of the association with a high risk of developing an infection, including tuberculosis (TB), depending on the geographic region and prevalence of the disease (2). Biological agents have a low immunosuppressive effect, but their mechanism of action may activate some targets that increase the risk for infections (3). On the other hand, many patients use non-steroidal anti-inflammatory drugs (NSAIDs) in order to ameliorate the symptoms, and in case of a higher degree of functional impairment, they also use glucocorticoids for long periods (4).

Vasculitis that is determined by systemic inflammation is responsible for the extra-articular manifestations of the disease (rheumatoid nodules or manifestations of cutaneous, pleural or pulmonary, cardiovascular, neurologic, ocular, hematologic, or digestive nature) (5). Pulmonary manifestations of the disease may include parenchymal rheumatoid nodules (which can excavate), interstitial lung disease (usual, non-specific, acute lymphocytic interstitial pneumonia or organizing pneumonia), pulmonary fibrosis, airway disease (bronchiolitis or bronchiectasis), pleural disease (effusion, pleuritis, pneumothorax, or empyema), Caplan syndrome, vascular disease (secondary pulmonary hypertension), toxicity caused by drugs used to treat the disease, infection, or amyloidosis. Pulmonary nodules are a frequent manifestation of RA, especially in males, smokers, and those with high levels of rheumatoid factor (RF) (5, 6).

Tuberculosis is a disease caused by *Mycobacterium tuberculosis* that can affect any organ. The infection can be inactive or latent, but only approximately 10% of people with latent or inactive infection develop active infection. The major site of infection is the lungs, and lymph nodes and the pleura are the most frequent extrapulmonary sites of infection (7). Drugs used in the treatment of RA represent an increasing risk factor for the reactivation of latent tuberculosis infection (LTBI) (8).

Herein, we present a case report to highlight the importance of screening in the context of a chronic long-term immunosuppressive therapy, particularly when radiological findings can include the pulmonary manifestations of an underlying rheumatological disease, can be secondary to the treatment, or can appear secondary to an infection such as tuberculosis.

## 2 Case report

We present the case of a 38-year-old male smoker with 30 pack-years of smoking, chronic alcohol consumption, and rheumatoid arthritis who was admitted to our hospital for respiratory failure with an oxygen saturation of 85% despite receiving 3 liters of supplemental oxygen per minute. He was also diagnosed with subcutaneous emphysema in the right laterothorax, which extended to the cervical region and face, and severe dyspnea. In addition, a clinical examination showed swollen finger joints, with limited finger movement, rheumatoid subcutaneous nodules, and severe pain at palpation and mobilization.

Within 9 months before admission to our hospital, the patient experienced weight loss and started to develop leucocytosis caused by neutrophilia. Five days earlier, the patient was admitted to a local hospital with an acute onset of cough and dyspnea. During the period of hospitalization, despite great efforts, he developed severe dyspnea and thoracic pain, with a pO_2_ of 26 mmHg, a pCO_2_ of 35.2 mmHg, a pH of 7.4, an SO_2_ of 48%, and a lactate level of 1.85 mmol/L in the arterial blood sample. Chest radiography confirmed the diagnosis of a right pneumothorax, following which a chest tube was inserted (Figure 1). A suspicion of pulmonary tuberculosis and an evolution of subcutaneous emphysema complicated the management of the case, and therefore, the patient was referred to our clinic.

**Figure 1:**
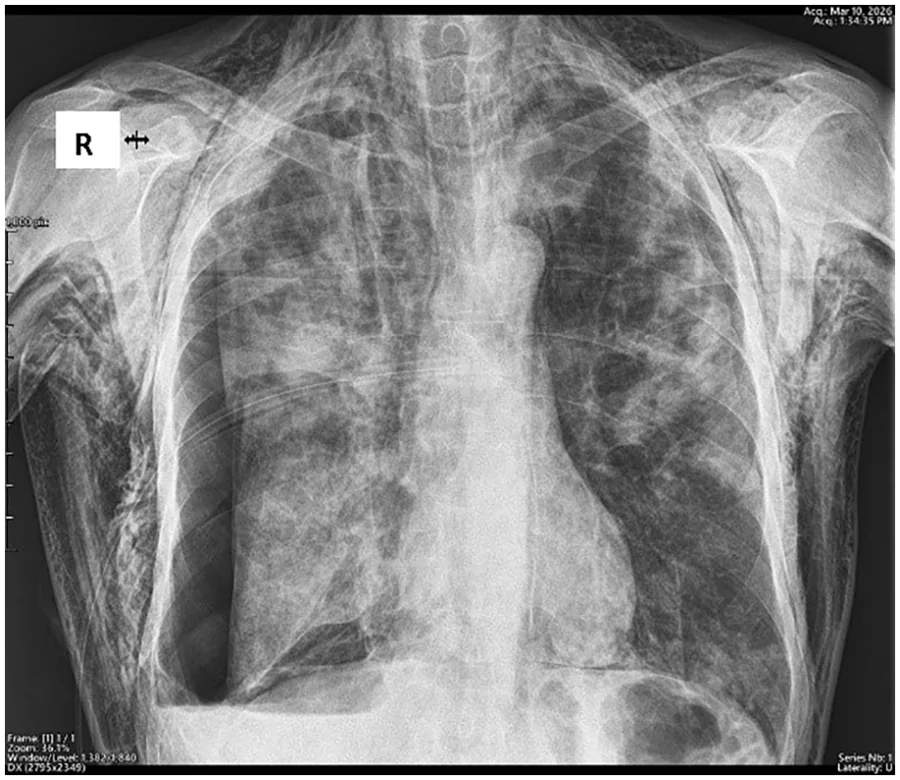
Chest radiography at admission showing a right pneumothorax with a chest tube and severe subcutaneous emphysema.

The clinical history of the disease began in 2021 when the patient was diagnosed with RA, with severe clinical manifestations including chronic pain, swelling, and redness of multiple articulations, especially the extremities, with positive RF levels (serologic value, 66.3 UI/mL; normal value, <30 UI/mL) and anticyclic citrullinated peptide antibody levels (anti-CCP; serologic value, 16.8 U/mL; normal value < 5 U/mL). Methotrexate treatment was initiated at that time. In addition, the patient received methylprednisolone intermittently and in different dosages, vitamin D3 (2000 UI), calcium, and different types of NSAIDs. He also received 20 mg of leflunomide per day since November 2025 and ended it on his own after 1 year. Despite the treatment, he had permanent symptoms, with episodes of exacerbations and elevated inflammatory marker levels (erythrocyte sedimentation rate and C-reactive protein) and RF values.

Upon admission to our clinic, a laboratory analysis of the blood samples showed leucocytosis (23.12×10^3^/μL; normal range (*N*) = 3.90–10.9 × 10^3^/μL) caused by neutrophilia (21.61×10^3^/μL; *N* = 1.8–6.98 ×10^3^/μL), inflammation with an erythrocyte sedimentation rate of 59 mm/h (*N* = 2–15 mm/h) and a C-reactive protein level of 296 mg/L (*N* = 0–5 mg/L), anemia with a hemoglobin level of 8.1 g/dL (*N* = 13.5–16.9 g/dL), and hypoalbuminemia (2.23 g/dL; *N* = 3.97–4.94 g/dL). Table 1 summarizes the most important blood parameters evaluated both before and during the spitalization.

**Table 1:** Evolution of blood parameters before and after hospitalization.

Parameter	Normal range	7 days b.a.	1 day b.a.	Day 1	Day 2	Day 3	Day 14	Day 25	Day 28	Day 61
Leucocytes	3.9-10.9×10^3^/µL	16.49	26.07	23.12	27.09	11.46	12.04	12.73	13.74	10.56
Neutrophils	1.8-6.98×10^3^/µL	13.50	24.92	21.61	24.85	10.07	7.52	7.75	9.94	6.76
ESR	2-15 mm/h	-	-	-	59	48	-	113	-	98
CRP	0-5 mg/L	122	244.3	296	185	112	-	147	>120	-
Hemoglobin	13.5-16.9 g/dL	8.7	8.6	8.3	8.4	8.1	6.9	6.3	7.1	7.6

ESR, erythrocyte sedimentation ratio; CRP, C-reactive protein; b.a., before admission.

Pleural fluid collected from the chest tube was an exudate with adenosine deaminase (86.8 U/L; *N*, 0–33 U/L), lactate dehydrogenase (2,186 U/L), glucose (3.48 mg/dL), and serum (122 mg/dL). GeneXpert was not available at that time, and no acid-fast bacilli were found via microscopy, with the culture contaminated.

Thoracic computed tomography showed bilateral pneumothorax, pneumomediastinum with severe associated subcutaneous emphysema, right pleural effusion, and multiple lesions suggestive of pulmonary tuberculosis (Figure 2).

**Figure 2:**
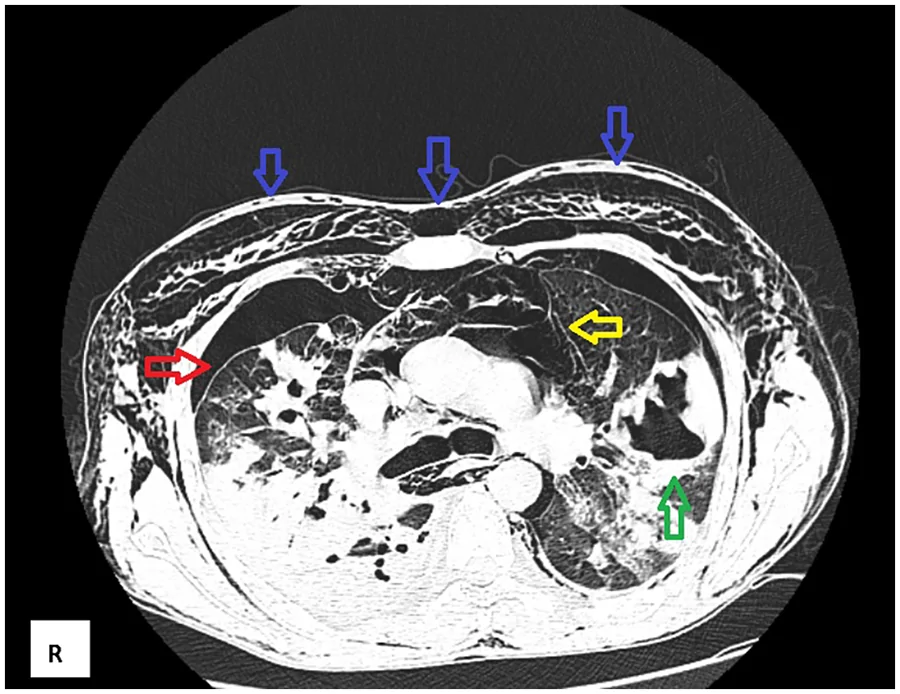
Thoracic computed tomography at admission, with a bilateral pneumothorax (red arrow), pneumomediastinum (yellow arrow), cavitary lesions (green arrow), and severe subcutaneous emphysema (blue arrows).

The patient was admitted to the ICU, a chest tube was inserted, and active drainage was initiated. After clinical stabilization, bronchoscopy was performed and the bronchial aspirate was sent to analysis. The analysis result was positive for acid-fast bacilli. The GeneXpert result was detected high with no resistance to rifampicin, and the antibiogram showed sensitivity to isoniazid and rifampicin. Standard antituberculosis treatment with weight-adjusted doses was initiated: a standard regimen with isoniazid (H; 300 mg daily), rifampicin (R; 600 mg daily), pyrazinamide (2,000 mg daily), and ethambutol (1,600 mg daily) for 2 months, followed by HR (300 and 600 mg, respectively) 3 days per week for another 4 months. For joint pain, the patient received non-steroidal anti-inflammatory drugs and methylprednisolone.

The clinical history of the disease and treatment are summarized in Table 2.

**Table 2:** Medical history and treatment received before and during hospitalization.

History					Admission to our clinic				Discharge
PR diagnosis			Weight loss, leucocytosis, and neutrophilia	Hospitalized for respiratory symptoms	Respiratory failure				
2021	November 2024	November 2025	June 2025	4 March	9 March 2026	10 March	12 March	3 April	7 May
	Leflunomide								
Methotrexate									
					Chest tube drainage				
							Treatment for tuberculosis		
Corticotherapy in different dosages and intermittent ones									

PR, polyrheumatoid arthritis.

The evolution was favorable, with good tolerance for the antituberculosis treatment and with slow and passive regression of the subcutaneous emphysema. Because of hypoalbuminemia, the patient developed significant bilateral leg edema, for which he received a low dose of diuretic treatment. After 1 month of admission, the patient accidentally removed the drain tube, following which he was monitored for 2 weeks with chest X-rays, remaining with a small lateral right-sided hydropneumothorax (Figures 3, 4). During the period of hospitalization, the patient’s major complaints were regarding the permanent pain experienced in the swollen joints, which was inconsistently reduced by the treatment.

**Figure 3:**
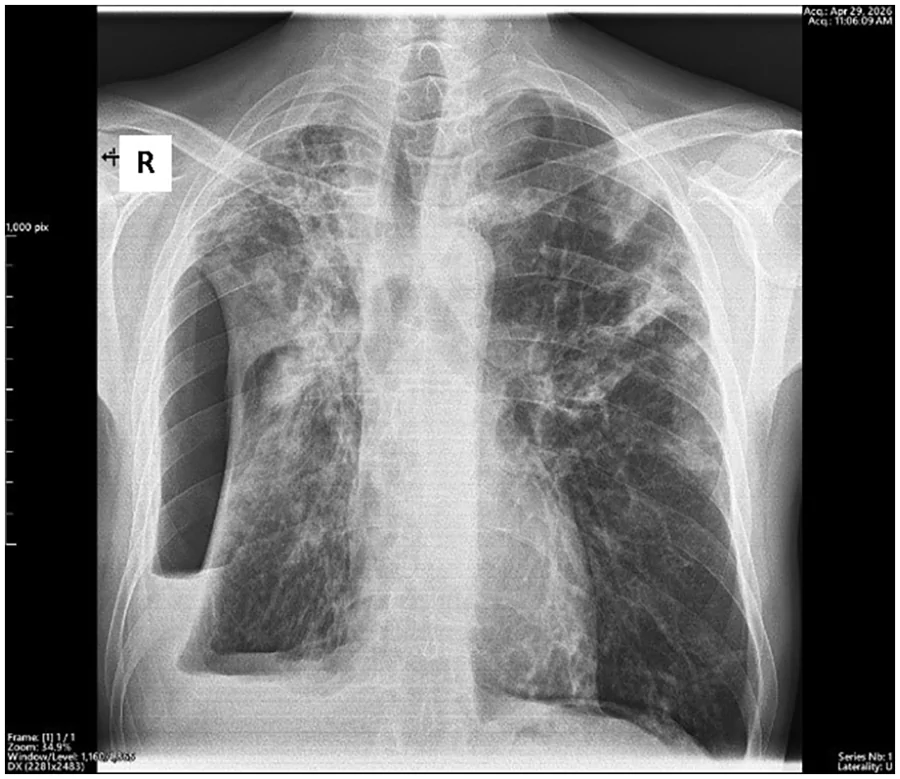
Chest radiography after 1.5 months of treatment (after the accidental removal of the chest tube).

**Figure 4:**
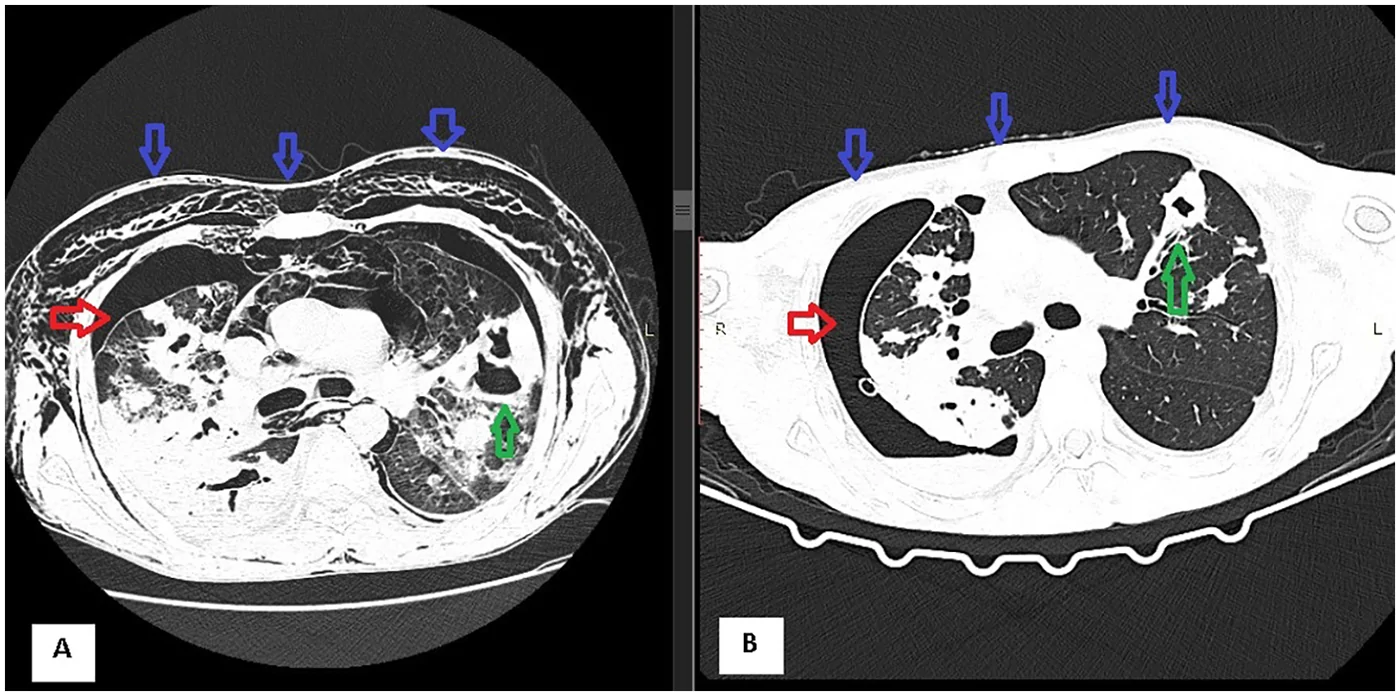
Thoracic computed tomography at admission **(A)** and after 1 month of treatment **(B)** showing a pneumothorax (red arrow), subcutaneous emphysema (blue arrows), and pulmonary lesions (green arrow).

We had scheduled a rheumatological examination to evaluate and manage treatment for the patient, but before doing so, the patient asked for discharge from the hospital and chose to continue treatment and investigations in a hospital near his home. At discharge, his blood samples showed normal leucocyte and neutrophil counts, inflammation with an erythrocyte sedimentation rate of 98 mm/h, and anemia with a hemoglobin value of 7.6 g/dL.

## 3 Discussion and literature review

According to the WHO, TB is a major health concern, is considered the world's leading cause of death caused by a single pathogen, and is the 10th leading cause of death globally (9). Romania is one of the countries with the highest incidence rate of tuberculosis in Europe. According to the Global Tuberculosis Report from 2025, the global incidence in 2024 was 131 cases per 100,000 population, and Europe had 1.9% of the total number of cases. More than half of the new cases were men (54%), followed by women (35%) and children (11%). Romania is classified under category 4 (50–99 incident cases per 100,000 population in 2024) from a total of six categories (9, 10).

The clinical diagnosis of active pulmonary tuberculosis is based on symptoms such as fever, cough with or without expectoration, weight loss, dyspnea, and hemoptysis (for a long duration with no improvement by antibiotic therapy) and bacteriological analysis, with the detection of bacilli using genetic tools or via direct microscopic analysis of the culture. Chest radiography is an important investigation technique, with high sensitivity and low specificity, that can show cavities and infiltrative or fibrotic lesions (11). A diagnosis of extrapulmonary tuberculosis is suspected based on the symptoms and depending on the organ affected and is confirmed via a histopathological examination or with positive culture if available (biopsy material should not be placed in formaldehyde because it destroys the mycobacteria) (12).

Inactive tuberculosis or latent infection is the asymptomatic infection with *M. tuberculosis*, without clinical, radiological, or bacteriological findings, and is therefore not contagious. Diagnosis is based on the innate response to bacterial antigens, and the tuberculin skin test (Mantoux test) and interferon gamma release assay (Quantiferon-TB Gold, Gold plus, or TB spot) were used. The purpose of identifying latent infection is to stop the development of an active one, and it is recommended in certain cases and situations given as follows: contacts of the diagnosed patients; immigrants from countries with high incidence rates of the disease; vulnerable population; chronic diseases such as silicosis, diabetes mellitus, or hemodialysis; before transplantation; and patients who require biological therapies or may be considered in situations that need prolonged treatment with corticosteroids or chemotherapy. If the test result is positive for infection in any of the above cases and situations, it is mandatory to start chemoprophylaxis (13). On the other hand, none of the ELISA-interferon gamma release assay (IGRA) screening tests can differentiate active from latent tuberculosis with 100% accuracy, and all situations need clinical judgment (14).

It is estimated that 2–3 billion people have LTBI and that a percentage of them (approximately 5%–10%) can develop active disease. In cases of patients with RA, the risk is 4 times higher than that in the general population (14, 15). The risk in patients with a prior history of infection or LTBI is 14.9, which is 2.58 times higher (16).

The screening protocol for LTBI includes assessment of the clinical history of exposure to *M. tuberculosis*, the tuberculin skin test, IGRA, and a chest radiography, with IGRA being the most useful method. In areas with an intermediate or high incidence, during the RA treatment, it is recommended to be done at 3 or 6 months for detecting reactivation and then after 18 months for detecting an active new infection. A study from Taiwan showed that the disease diagnosed after 3 months of treatment may be a consequence of LTBI reactivation, and in patients diagnosed after 20–24 months, it is more likely to be a new infection (14, 17, 18). Apart from the screening, some preventive measures need to be taken because, in some patients, the early occurrence of disease was diagnosed after 1 year of TNF alpha treatment (19).

Screening tests such as QuantiFERON-TB Gold In-Tube and IGRA have the same utility and have high specificity, but they can identify prior TB infection, leading to a low sensitivity. In addition, in patients with a decreased total number of lymphocytes or CD2^+^, both methods can lead to high rates of false-negative results (20).

The risk of patients receiving non–anti-TNF-targeted biologic agents is negligible, and in those patients, LTBI screening is not necessary (21). In Romania, screening includes a QuantiFERON test for biological agents such as TNF alpha, but it is not recommended in case of methotrexate use (13).

A study including 275 patients with RA treated with biological agents showed that 13.6% of them presented a conversion to at least one screening test during the therapy (22).

There is some evidence that there is a causal relation between RA and TB. Studies on mice showed that the *M. tuberculosis* infection can increase serum IL-6 levels and the expression of TLR2 receptors, accelerating the risk for RA development. In addition, an immune reaction to the proteins of the bacillus can produce reactive arthritis (23). Furthermore, the use of TNF alpha agents increases the risk for extrapulmonary and disseminated tuberculosis to 60% and 26%, respectively, by affecting the immune response of TNF against the bacillus. The mechanism involves increasing the capacity of TNF to enhance the antibacterial macrophage autophagy on the pathogen, destroying and forming granulomas, thereby preventing the systemic spread of the infection (21, 24). Furthermore, it promotes the maturation of phagosomes and induces the antimicrobial peptide production, producing reactive oxygen and nitrogen species, and facilitates the recruitment of immune cells at the infection site. In addition, TNF alpha activates B cells and cytotoxic CD8^+^  T cells. The use of anti-TNF alpha agents produces a disruption of the immune response, leading to dissemination and progression of the disease (25).

Another study conducted on mice showed that the production of IL-6 and IL-17 by neutrophils was linked to the protection against TB infection. IL-17 is required for protection against the bacillus but not for the control of the bacterial infection, and the study found that elevated serum levels of this interleukin induced by the infection increased the severity of arthritis (26).

The risk of patients with RA developing tuberculosis is 3.2  times higher than those without RA developing the disease, 10 times higher in males, and more frequent at young ages (from 20 to 39 years). This risk is also increased by the coexistence of other conditions such as underweight, diabetes mellitus, arterial hypertension, chronic renal disease, and respiratory disease (27–29). In the event of airway disease secondary to RA, this condition may be a possible cause of patients developing non mycobacterium tuberculosis (NTM) infection (30).

In an article from 1952, a case of a 17-year-old female with polyarthritis determined by tuberculosis is presented, and there is a theory that a small bacteremia secondary to bacillary infection may determine articular inflammation or even that the articular pathology produces a sensitivity of the organism upon bacillus contact (31). The association between tuberculosis and RA has been known since 1912 and was then recalled in 1955 when the term “rheumatoid diathesis” appeared, referring to a genetic or constitutional predisposition to inflammatory diseases and stating that the pulmonary lesions that appear in RA may precede the onset of arthritis by several years (5, 32, 33). Some people believed that rheumatoid arthritis was a form of tuberculosis (34).

A study conducted on mice showed that MTG (*M. tuberculosis* group organism) RNA sequences were identified in in synovial tissue of a high proportion of cases with RA, even in case of arthritis but with a lower frequency, and were absent in healthy synovial tissue (35).

The majority of patients with RA develop pulmonary tuberculosis (84%), and the most frequent extrapulmonary site is represented by the lymph nodes (36).

The risk of patients with RA developing pulmonary tuberculosis increases from 2 to 10 times in those who receive biological agents, and it develops in the first year of the treatment (37). Data from the European and American Registries showed that 25%–56% of the patients diagnosed with RA received biological agents (38). This risk may be reduced by performing LTBI screening and initiating prophylaxis before initiating therapy. From a total number of 2,429 patients with RA or other articular diseases who received anti-TNF alpha treatment, six developed active tuberculosis (19).

A 58-year-old female with RA developed disseminated pulmonary tuberculosis after the initiation of infliximab, without any prior LTBI screening (39), and a 72-year-old male with a 25-year history of RA developed pulmonary tuberculosis 1 month after the administration of infliximab (40). From all the biological agents used, abatacept is an exception, not being contraindicated in case of active mycobaterial disease, and may be used concomitantly with the tuberculostatic treatment. A 75-year-old male presented with active tuberculosis after the administration of abatacept and developed a paradoxical reaction and an exacerbation of the rheumatological disease after the cessation of treatment. A decision was made to concomitantly administer the treatment for both diseases, and the result was a favorable one (41).

Sometimes, even if the screening result is positive and prophylaxis is done correctly, mycobacterial infection can occur after biological agents are administered (42). In other cases, the risk depends on the dose (e.g., tofacitinib, a JAK inhibitor) (38).

The risk exists even in the absence of biologic therapy. For example, a 45-year-old male with articular involvement and a diagnosis for 8 years, who was treated for 3 years with NSAIDs and then with methotrexate and hydroxychloroquine, developed pulmonary tuberculosis, with no prior LTBI screening (5).

Extrapulmonary tuberculosis occurs in 15%–20% of the total number of cases, and any organ can be involved in this infectious disease. Cases of osteoarticular localization in patients with RA have been reported: arthritis in a 72-year-old female with knee tuberculosis after she received treatment with etanercept and adalimumab for 2 years (43); a 61-year-old female with a history of two episodes of pulmonary tuberculosis who developed Pott’s disease after DMARDs and biologic therapy for RA and Sjogren’s syndrome, respectively (44); and a 67-year-old male with tuberculous synovitis complicated with carpal tunnel syndrome, which was confirmed by a positive culture for *M. tuberculosis* and nerve conduction studies, who received treatment with methotrexate and hydroxychloroquine (45). A differential diagnosis of arthritis manifestation from rheumatological disease or infectious disease is difficult; for example, an 83-year-old male with RA developed *M. avium* sacroileitis and, at this age, both conditions are likely to be encountered in the geriatric population (46).

Other sites of infection are also described in the literature for patients with RA treated with TNF alpha antagonists: a case of primary nasal tuberculosis on a patient (47), a case of tonsillar tuberculosis diagnosed histopathologically and microbiologically after 8 months of treatment (33), a case of compressive tuberculous pericarditis (48), and a case of polyserositis with bilateral pleural effusion and ascites, which was tested positive for tuberculosis from the gastric fluid with elevated levels of ACE in a 55-year-old female (25). Angiotensin converting enzyme (ACE) levels are higher in pleural effusion with RA or TB etiology, as a consequence of local immune cellular response, and along with beta 2 microglobulin, it has a sensitivity of 91% and a specificity of 86% vs. 55% in the diagnosis of RA (49). A 69-year-old female was diagnosed with mycobacterium-associated lobular panniculitis, which was first considered a rheumatoid nodule, and differential diagnosis by PCR or acid-fast staining must be performed routinely from tissue biopsy in case of skin lesions that are indurated (50).

There are some controversial data regarding the use of methotrexate, and with regard to prednisone, no significant difference is linked to the maximum dose and the duration of treatment before the diagnosis of active tuberculosis (36). The long-term use of corticosteroids decreases the resistance of the organism against infection, and the recommendation is not to use them in case of active disease and with extreme caution in cases of LTBI until further investigations confirm their safety (51). One study found that a medium dose of glucocorticoids for a 1-year treatment is an independent risk factor for tuberculosis infection (52).

A study that included 951 patients with RA showed that there were no significant differences in terms of risk in patients who used adalimumab or etanercept, that sulfasalazine had a protective role through the antibacterial effect of sulfonamides, and that no risk was associated with the use of golimumab and tsDMARDs (15).

The specificity of the case that we presented here is attributed to a rare and unusual complication of tuberculosis, particularly pneumomediastinum and bilateral pneumothorax, in a young patient with an underlying disease, which can be considered a risk factor by itself or by the treatment administered for rheumatological symptoms.

## 4 Conclusions

We presented a case of severe complications derived from the association between rheumatoid arthritis treated with immunosuppressive medication and secondary pulmonary and possible pleural tuberculosis, which resulted in the development of a bilateral pneumothorax and pneumomediastinum. This case is a reminder of the importance of LTBI and also periodical screening of tuberculosis in countries with high incidence of the disease, especially when the initiation of an immunosuppressive chronic treatment is mandatory and which lasts for a long duration. In addition, even more important is the detection of an active disease with the indication of performing a chest radiography and sputum tests in the case of a patient with suggestive symptoms, weight loss, and chronic cough.

## Data Availability

The raw data supporting the conclusions of this article will be made available by the authors, without undue reservation.
